# Biallelic Germline *BRCA1* Frameshift Mutations Associated with Isolated Diminished Ovarian Reserve

**DOI:** 10.3390/ijms252212460

**Published:** 2024-11-20

**Authors:** Anne Helbling-Leclerc, Marie Falampin, Abdelkader Heddar, Léa Guerrini-Rousseau, Maud Marchand, Iphigenie Cavadias, Nathalie Auger, Brigitte Bressac-de Paillerets, Laurence Brugieres, Bernard S. Lopez, Michel Polak, Filippo Rosselli, Micheline Misrahi

**Affiliations:** 1 Genome Integrity and Cancer, CNRS UMR9019, Université Paris-Saclay, Gustave Roussy, 94805 Villejuif, France; anne.helbling-leclerc@universite-paris-saclay.fr (A.H.-L.); filippo.rosselli@gustaveroussy.fr (F.R.); 2 Service d’Endocrinologie, Gynécologie et Diabétologie Pédiatrique, APHP Hôpital Universitaire Necker Enfants Malades, 75743 Paris, France; marie.falampin@aphp.fr (M.F.); maud.marchand@ch-douai.fr (M.M.); iphigenie.cavadias@aphp.fr (I.C.); michel.polak@aphp.fr (M.P.); 3 Centre de Référence Maladies Rares-CRMR des Pathologies Gynécologiques Rares, 75743 Paris, France; 4 Unité de Génétique Moléculaire des Maladies Métaboliques et de la Reproduction, Laboratoire de Référence Pour les Infertilités Génétiques, APHP Hôpitaux Universitaires Paris-Saclay, Faculté de Médecine Paris Saclay, Hôpital Bicêtre, 94275 Le Kremlin-Bicêtre, France; abdelkader.heddar@gmail.com; 5 Département de Cancérologie de L’enfant et de L’adolescent, Gustave Roussy, Université Paris Saclay, 94805 Villejuif, France; lea.guerrini-rousseau@gustaveroussy.fr (L.G.-R.); laurence.brugieres@gustaveroussy.fr (L.B.); 6 Département de Biologie et de Pathologie Médicales, Gustave Roussy, 94805 Villejuif, France; nathalie.auger@gustaveroussy.fr; 7 Département de Biologie et Pathologies Médicales et U1279 INSERM, Gustave Roussy, Université Paris-Saclay, 94805 Villejuif, France; brigitte.bressac@gustaveroussy.fr; 8 Faculte de Medecine, INSERM 1016, UMR 80104 CNRS, Institut Cochin, Université de Paris-Cité, 24 Rue du Faubourg ST Jacques, 75014 Paris, France; bernard.lopez@inserm.fr; 9 Faculté de Santé, Université de Paris, 75006 Paris, France; 10 Groupement de Coopération Sanitaire-GCS SeqOIA, Référent Clinicien Préindication Insuffisance Ovarienne Primitive and Plan France Médecine Génomique 2025, 78 rue du Général Leclerc, 94270 Le Kremlin-Bicêtre, France

**Keywords:** *BRCA1* mutation, diminished ovarian reserve, primary ovarian insufficiency, DNA repair, meiosis, Fanconi anemia, genetic counseling

## Abstract

The use of next-generation sequencing (NGS) has recently enabled the discovery of genetic causes of primary ovarian insufficiency (POI) with high genetic heterogeneity. In contrast, the causes of diminished ovarian reserve (DOR) remain poorly understood. Here, we identified by NGS and whole exome sequencing (WES) the cause of isolated DOR in a 14-year-old patient. Two frameshift mutations in *BRCA1* (*NM_007294.4*) were found: in exon 8 (c.470_471del; p.Ser157Ter) and in exon 11 (c.791_794del, p.Ser264MetfsTer33). Unexpectedly, the patient presented no signs of Fanconi anemia (FA), i.e., no developmental abnormalities or indications of bone marrow failure. However, high chromosomal fragility was found in the patient’s cells, consistent with an FA diagnosis. RT-PCR and Western-blot analysis support the fact that the c. 791_794del *BRCA1* allele is transcribed and translated into a shorter protein (del11q), while no expression of the full-length BRCA1 protein was found. DNA damage response (DDR) studies after genotoxic agents demonstrate normal activation of the early stages of the DDR and FANC/BRCA pathway. This is consistent with the maintenance of residual repair activity for the del11q BRCA1 isoform. Our observation is the first implication of bi-allelic *BRCA1* mutations in isolated ovarian dysfunction or infertility in humans, without clinical signs of FA, and highlights the importance of BRCA1 in ovarian development and function.

## 1. Introduction

Infertility is a major public health problem that affects 17.5% of couples in developed countries [1]. Primary ovarian insufficiency (POI) and diminished ovarian reserve (DOR) are major pathological situations causing female infertility. POI refers to the premature cessation of ovarian function before the age of 40 years, with amenorrhea or spaniomenorrhea for over 4 months and elevated follicle-stimulating hormone (FSH) plasma levels ≥25 IU/L on at least two occasions [2,3]. Resumptive POI with ovarian activity and even rare spontaneous pregnancies have also been described, respectively, in 24% and 4.4% of cases [4]. DOR can be defined by the criteria of the third group of the Poseidon classification (Patient-Oriented Strategies Encompassing Individualize D Oocyte Number) with anti-Müllerian hormone (AMH) plasma levels of <1.2 ng/mL and/or an antral follicle count (AFC) of <5 before the age of 35 years [5]. Patients with DOR represent 10% of the women undergoing assisted reproductive technology (ART), in particular, in vitro fertilization (IVF) [3].

The causes of POI are very diverse [3]. POI may be due to an altered establishment of the follicular pool antenatally, altered follicular growth after puberty or increased follicular atresia during prenatal or postnatal life [3]. POI can either be isolated or syndromic, including other organ defects. Isolated POI can be due to iatrogenic causes (chemotherapy, radiotherapy, or ovarian surgery), viral infections, and autoimmune diseases (associated with Hashimoto thyroiditis). About 30% of POIs are familial, strongly supporting the existence of genetic causes. Chromosomal anomalies account for up to ~15% of patients (Turner syndrome 45 XO with or without mosaicism or other rearrangements of the X chromosome), but ~70% of POI causes remained unknown. Genetic causes may also be involved but only the study of the fragile X messenger ribonucleoprotein (FMR1) premutation was routinely performed, and it turned out positive in 3–5% of patients [3]. The recent uses of next-generation sequencing (NGS) and whole exome sequencing (WES) have allowed a leap in knowledge, supporting a strong and heterogeneous genetic basis for POI [3,6].

We have recently shown in a large international cohort of patients with unexplained POI that a custom-targeted NGS-POI panel comprising all known responsible genes (nearly a hundred) allowed a high diagnostic yield of 29.3%, supporting a routine clinical diagnosis, and leading to personalized medicine. The major gene family was the meiosis/DNA repair gene family (37.4% of cases), followed by genes involved in follicular growth (35.4%) and in metabolism and mitochondrial functions (19%). Some genes may also be involved in ovarian development, immunity, and autophagy [7]. We have shown that 45.9% of patients may have or are at risk of developing associated comorbidities, requiring a comprehensive assessment by a multidisciplinary team [7].

Notably, many of the identified genes overlap with genes in which loss of function leads to genetic diseases associated with cancer, premature aging, genetic instability, and impaired DNA damage response (DDR) and repair [3,7,8]. This observation highlights a critical pathological role in POI for genes involved in replication and/or homologous recombination (HR), known to be involved in meiosis. Cells engaged in gametogenesis first undergo an expansive replicative phase. Successively, the meiotic process takes place and allows the mix of maternal and paternal genomic information in the mature gametes via two processes: HR-mediated exchange of DNA between homologous paternal and maternal chromatids, followed by two chromosome segregation steps, Meiosis I and II, that yield the haploid gametes. Therefore, the defects of genes involved in replication, DDR, and/or DNA repair are expected to have an impact on gametogenesis. Accordingly, we recently reported a patient with an isolated POI and a hypomorphic homozygous mutation in the breast cancer susceptibility gene type 2 (*BRCA2*), the encoded protein that participates in the HR process [9]. Rare hypomorphic biallelic *BRCA2* mutations have also been associated with Fanconi anemia (FA), a syndrome characterized by developmental abnormalities and bone marrow failure [10,11,12,13]. However, we identified a homozygous hypomorphic *BRCA2* mutation in a patient with isolated POI without signs belonging to an FA phenotype at the time of the diagnosis, i.e., without bone marrow failure or developmental abnormality. In agreement with this fact, we found that the mutated protein retained partial HR activity [9].

Mutations in more than 20 genes are known to cause FA and encode proteins primarily involved in DNA repair and HR that constitute the FANC/BRCA repair pathway, which inactivation results in chromosomal fragility and cellular hypersensitivity to DNA-damaging agents, particularly those inducing cross-links between DNA strands, such as mitomycin C (MMC) or inhibiting PARP1 activity [12]. FA is an inherited bone marrow failure syndrome (iBMFS) [14]. However, beyond its two major pathological traits, the BMF and the associated anemia/pancytopenia, FA patients can present additional pathological features, including skeletal, epithelial, endocrine, sensorial, immunological, and cognitive abnormalities. Somewhat surprisingly, 20–30% of the patients are symptomless before the development of their hematological abnormalities, which can be present at birth or appear later during infancy and adolescence up to adulthood [13]. Compared to individuals of the same age, FA patients are at risk for myelodysplastic syndrome (MDS), acute myeloid leukemia (AML), and head-and-neck cancers [13]. FA patients and mouse models often exhibit altered gametogenesis and infertility [11,15]. We have shown that the mutation of *FANCM*, another gene of the FA pathway, causes isolated POI [16]. The functionality of the FANC/BRCA pathway seems to be required for normal gametogenesis and fertility. On the other hand, mutations in a single protein of the pathway can affect gametogenesis and, potentially, DNA repair, leading to POI with [17,18,19,20] or without [9,21] a canonical FA syndrome.

Contrasting with POI, very few genetic causes of DOR have been described. DOR and POI might share, in part, common mechanisms. DOR could progress towards POI, and identifying the causes could be important in the personalized care of patients. DOR could be, in some cases, an early stage of POI.

Here, we describe a 14-year-old patient with an isolated DOR, carrying compound heterozygous frameshift mutations in the breast cancer susceptibility gene type 1 (*BRCA1*). *BRCA1* is also an FA-associated gene involved in HR at a stage preceding BRCA2 [22]. Unexpectedly, given her genetic state, the patient presents a mild phenotype with isolated DOR and no evidence of FA, i.e., no developmental abnormalities or (so far) bone marrow failure (BMF). However, cytogenetic studies revealed a high chromosomal fragility and susceptibility to DNA damage in cells compatible with a diagnosis of Fanconi anemia. This is in favor of hypomorphic mutations, which allow the expression of a mutated BRCA1 protein that maintains certain WT activities, and mitigates the pathological consequences of the mutation. Our observation is the first implication of bi-allelic *BRCA1* mutations in isolated ovarian dysfunction or infertility in humans, without clinical signs of FA, and highlights the importance of BRCA1 in ovarian development and function.

## 2. Results

### 2.1. Case Presentation

We report a female adolescent, born to a Caucasian Portuguese mother and a Brazilian father, who was first referred at the age of 14 to the endocrinology clinic of Necker Hospital (Paris, France) due to secondary amenorrhea, symptoms of hyperandrogenism, and high blood pressure.

**Figure 1:**
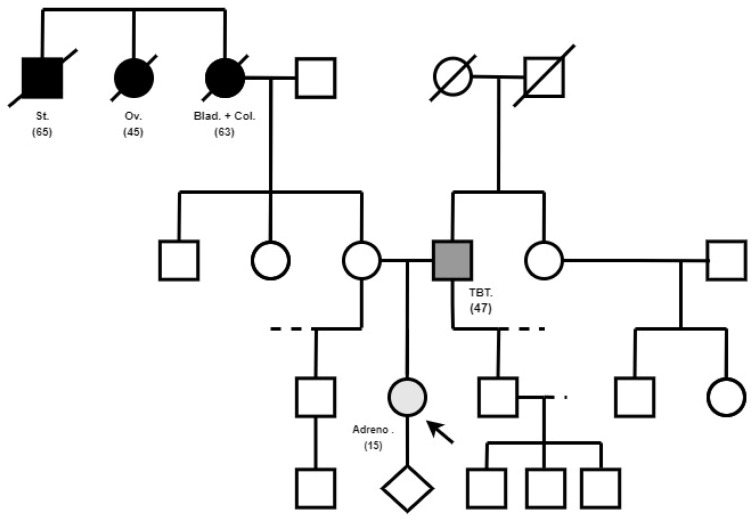
Clinical feature of the family of the proband. Cancer family history including age at the first diagnostic is reported. St. = stomach cancer, Ov. = ovarian cancer, Blad. = bladder cancer, Col. = colorectal cancer, TBT. = temporal benign tumor, Adreno. = adrenocortical adenoma.

Her personal medical story was unremarkable. She is the only child of an unrelated couple with two older healthy half-siblings and four nephews. Her maternal grandmother (deceased) had bladder and colorectal neoplasia at the age of 63, her maternal great uncle (deceased) had stomach cancer at the age of 65, and her maternal great aunt (deceased) had ovarian cancer at the age of 45 (Figure 1). The parents have no history of malignant tumors; however, her father has presented a temporal benign tumor at the age of 47. The patient’s mother is healthy; she is about 1.76 m height. The father’s height is unknown. Her paternal grandmother suffered from an undetermined congenital heart disease.

The patient had a spontaneous puberty with menarche at the age of 11 years, rapidly followed by primo-secondary amenorrhea after only one episode of menstruation. A progressive worsening of hirsutism and a deep voice associated with high blood pressure were observed. The Ferriman–Gallwey score, that allows for the clinical assessment of the degree of hirsutism in a woman, was of 28. The external genitalia were female without clitoridomegaly. Many café-au-lait spots were observed. Basal hormonal assays revealed extremely high plasma levels of dehydroepiandrosterone sulfate and testosterone with suppressed gonadotrophins. Plasma oestradiol levels were normal whereas AMH plasma levels were very low for her age (0.10 ng/mL). Abdominal ultrasounds and CT scans revealed a five centimeter tumor in the left adrenal gland. An evaluation of tumor extension was negative, and adrenalectomy was performed. A histopathological examination confirmed the diagnosis of an adrenocortical adenoma. No other treatment was indicated. One month after surgery, androgen levels returned to normal and she recovered spontaneous regular cycles. Regular ultrasounds since her operation have revealed no recurrence.

The patient was then referred to the Reference Center for Rare Gynecological Pathologies in Necker Hospital for heavy menstrual bleeding. Her gynecologic symptoms led to a screening for a bleeding disorder. They revealed a quantitative deficiency of von Willebrand factor. The diagnosis of von Willebrand disease type 1 was made by a hematologist and non-hormonal treatment was offered.

Plasma hormone levels were controlled on cycle day 3: gonadotrophins levels were elevated for the follicular phase normal range: FSH: 11.2 UI/L and LH: 12.8 UI/L, and the AMH level was still low at 0.10 ng/mL, suggesting a diminished ovarian reserve (DOR).

She used a combined oral contraceptive (COC) between the ages of 15 and 16.5 years. After COC was stopped, she experienced hot flashes and one 4 month episode of amenorrhea. Then, withdrawal bleedings were induced by progestins for a few months before she recovered spontaneous regular cycles.

Hormonal levels were regularly assayed between the ages of 16.5 and 19 years (Table 1), a period during which she had no hormonal contraceptive. Her gonadotrophins levels were fluctuant. Her FSH level reached 49 UI/L and 25 UI/L on two occasions at more than 4 weeks apart, leading to the diagnosis of POI. After four months of amenorrhea, she recovered an ovarian function with spontaneous regular menses. Moreover, she underwent a few transvaginal ultrasounds. Her ovaries were small (2 cm^2^ for right and 2.4 cm^2^ for left ovaries); antral follicle count was one to three on the right and one to four on the left ovary. Fertility preservation counseling was offered but no procedure was engaged.

In order to document the POI, first-line investigations did not reveal any cause: karyotype was 46 XX, there was no *FMR1* premutation, and thyroid autoantibodies were negative. Genetic studies were then performed in the molecular genetics laboratory of Bicêtre Hospital: a POI panel comprising 88 genes [7] did not reveal any pathogenic or likely pathogenic variant in a known POI gene. Because of numerous café-au-lait spots and benign adrenocortical adenoma, genetic investigations for a cancer predisposition syndrome were performed at the Gustave Roussy Cancer Institute. No mutation was found in the *NF1*, *SPRED1*, or *TP53* genes. The benign adrenocortical adenoma did not display microsatellite instability (MSS). A panel of 45 genes (see Methods) including among others HR genes (*ATM-BARD1-BRCA1-BRCA2-BRIP1-CHEK2-NBN-PALB2-RAD51B-RAD51C-RAD51D*), mismatch repair (MMR) genes (*MLH1-MSH2-MSH6-PMS2*) as well as *TP53* was studied at Gustave Roussy Institute [23]. It revealed compound heterozygous pathogenic variants of *BRCA1* (described below).

In this context, the follow-up strategy to manage the high risk of cancer in this young patient was discussed in a multidisciplinary team including oncologists and geneticists. Annual breast magnetic resonance imaging (MRI) from the age of 20 onward and regular evaluations together with otorhinolaryngologist, gynecologist, proctologist, dermatologist, and hematologic surveillance with a basal myelogram and blood counts every 3 or 4 months were recommended. A discussion with the patient about prophylactic measures such as mastectomy was started. The parents refused any blood assay and genetic counseling.

The patient is now 19 years old. Her final size is 158 cm for 61 kg with a body mass index of 24 kg/m^2^. Her head circumference is normal at 56 cm. Regular blood tests did not reveal any sign of cytopenia. She had regular menstrual cycles without any symptom of estrogenic deprivation and presented isolated DOR. She experienced for months of frequent menstrual bleeding (<21 days) before she spontaneously got pregnant. She was in a stable relationship for six months and was having unprotected intercourses. She did not wish to use contraceptives since she was followed by a fertility clinic in an attempt to preserve her fertility. Her first trimester ultrasound was perfectly normal and first trimester screening for down syndrome was negative. She is now in her second trimester and the pregnancy is going well.

### 2.2. Identification of the BRCA1 Mutations

**Figure 2:**
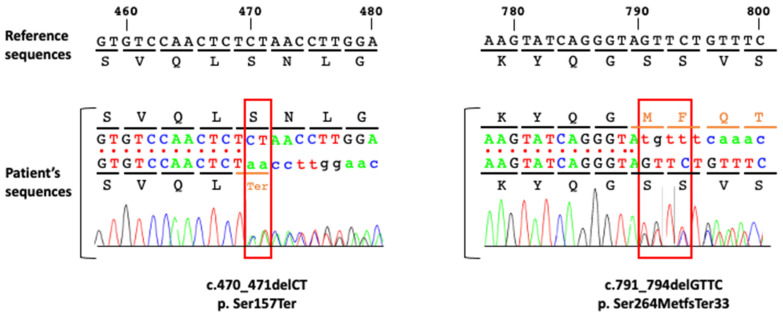
Confirmation of the two *BRCA1* variants identified in the patient by Sanger Sequencing. The two variants in the patient were confirmed by Sanger sequencing as shown by the two electropherograms centered around the first variant (c.470_471del; p.Ser157Ter) located in exon 8 (left panel) and the second variant (c.791_794del, p.Ser264MetfsTer33) located in exon 11 (right panel). The deleted nucleotides (two and four nucleotides respectively) are highlighted by a red rectangle.

Targeted NGS analysis and WES were performed in the proband and allowed for the identification of two variants in *BRCA1*, yielding two frameshift mutations in exon 8: *BRCA1* (NM_007294.4:c.470_471delCT (p.Ser157Ter) and in exon 11: *BRCA1* (NM_007294.4:c.791_794delGTTC (p.Ser264MetfsTer33) (Figure 2).

Both *BRCA1* variants are predicted to be pathogenic according to the ACMG classification and are pathogenic in Clinvar. *BRCA1* appears to be the only candidate gene found by WES to be involved in the patient’s POI. All other known genes causing POI were normal.

The c.791_794del variant has a low 0.00000801 frequency in the gnomAD exome and is absent in the gnomAD genome, while the second variant (c.470_471delCT) is absent in both databases. The two variants are absent in the Catalogue Of Somatic Mutations In Cancer (COSMIC v98, released 23 May 2023). Both mutations have already been described separately and have been associated with infiltrating ductal carcinoma [24], breast, and ovarian cancer [25,26,27,28] in heterozygous carriers from several countries and ethnic groups [25,26,27].

Family segregation could not be performed because the patient had no contact with the paternal branch of the family for several years. The mother refused genetic analysis.

### 2.3. Cytogenetic and Growth Studies of Patient’s Cells

As BRCA1 is involved in the FANC/BRCA DNA repair pathway, we performed cytogenetic analyses of the patient’s immortalized lymphoblasts under basal unstressed conditions, in response to the DNA cross-linking agent (Mitomycin C, MMC at 150 and 300 nM), to which cells deficient for the FANC/BRCA pathway are known to be hypersensitive [29].

**Figure 3:**
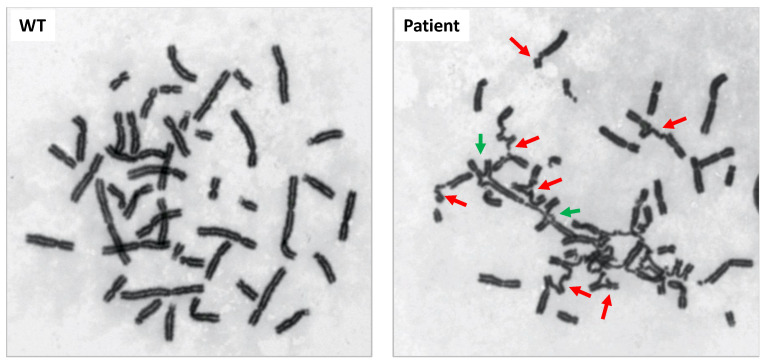
Chromosomal fragility in response to mitomycin C on metaphase chromosomes. Chromosomal breakages and radial figures are shown, respectively, by red and green arrows.

In untreated cells, 8% of the metaphases present chromosomes with DNA breaks compared to their absence in the metaphases of two non-affected healthy individuals. In the presence of 150 nM MMC, almost all the metaphases of the patient (96%) exhibit chromosomal aberrations, compared to only 2 to 14% in healthy cells (Table 2). Moreover, metaphases present extremely high levels of chromosomal breaks and radial figures, the latter being considered a hallmark of FA cells (Table 2, Figure 3).

**Figure 4:**
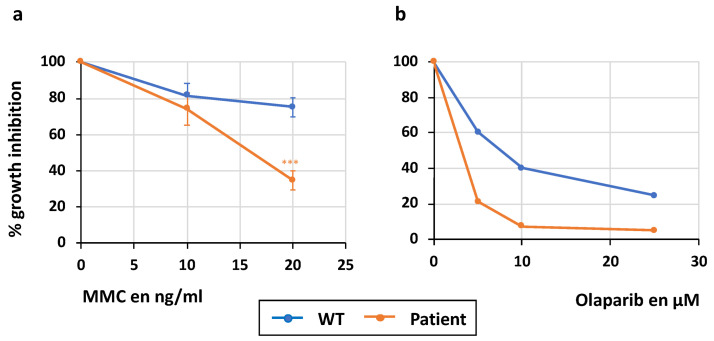
Survival curve after genotoxic treatment. (**a**) Survival curve after mitomycin C (MMC) and (**b**) Olaparib treatment during the 3 days. (n = 3 for MMC tests; mean ± SEM; GraphPad unpaired *t* test *** *p* < 0.001 and n = 1 for Olaparib test).

Consistent with a BRCA1 activity deficiency, the proliferation of the patient’s lymphoblasts is strongly affected by exposure to MMC or the PARP inhibitor (PARPi) Olaparib, known for its toxicity on BRCA1/2-deficient cells [30] (Figure 4).

Overall, our results are consistent with a diagnosis of FA for the patient, although no dysmorphia, microcephaly, BMF, anemia, or thrombocytopenia were observed at the time of our analysis. As monoallelic *BRCA1* mutations do not confer MMC sensitivity and FA cellular phenotype [31,32], we concluded that the identified mutations are in a trans, and that the patient is a compound heterozygous, having inherited a pathogenic allele of each parent.

### 2.4. BRCA1 Transcripts and Proteins Produced by the Mutated Patient’s Alleles

**Figure 5:**
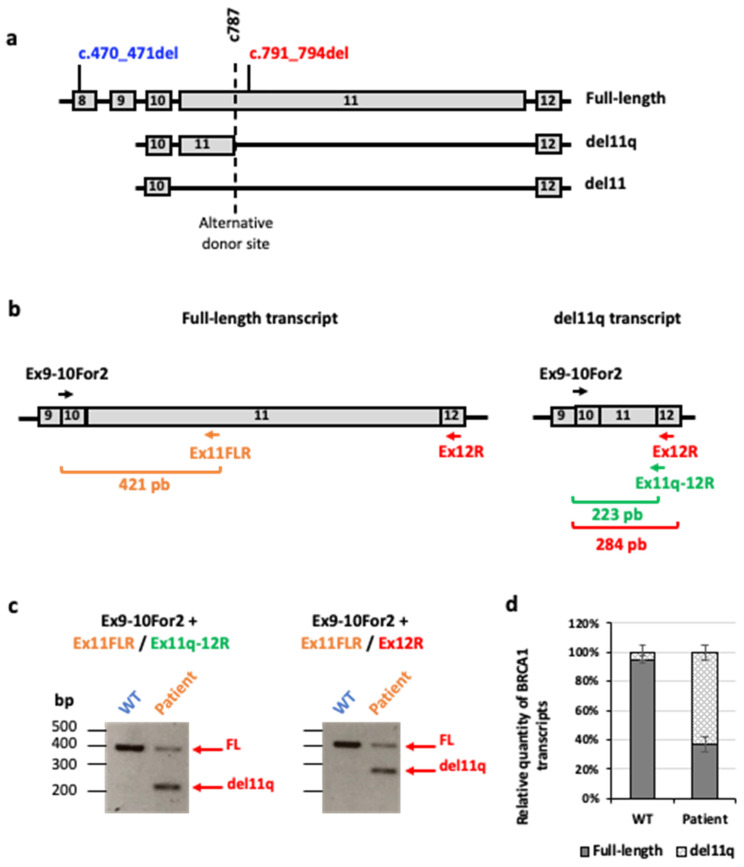
*BRCA1* transcripts analysis. (**a**) Partial genomic organization of *BRCA1* with both mutations identified in the patient’s DNA. The dashed line at *BRCA1* c.787 indicate an alternative splice site that yields an in-frame truncated transcript, *BRCA1* del11q [35]. (**b**) Localization of the primers and size of the amplicons generated to distinguish the full-length (FL) and the del11q *BRCA1* transcripts. (**c**) *BRCA1* transcripts: RT-PCR from lymphoblasts cells of a control (WT) and the patient using three pairs of primers amplifying the reference transcript (FL) or the del11q isoform. (**d**) Relative quantity of *BRCA1* transcript isoforms from three replicate experiments with two different amplicons for the full-length and del11q transcripts. (mean ± SEM).

*BRCA1* is known to produce different transcripts that coexist with a main isoform which contains all *BRCA1* exonic coding sequences [33]. Additionally, some *BRCA1* mutations have been associated with alternative transcripts isoforms. In particular, exon 11 can be completely deleted, leading to the del11 isoform, or partially deleted due to an alternative splice site at position c787, giving the del11q isoform that retains the first 117 nucleotides of the exon in frame [34]. The truncated protein derived from the del11q mRNA has a partial activity (on average 50%) [35]. The partial genomic organization (exons 8–12) of *BRCA1*, with the two mutations identified in the patient, the localization on DNA and the cryptic donor splice site are presented in Figure 5a as well as major transcripts isoforms.

To evaluate the relative expression of the full-length and splicing of the del11q isoform transcripts, we performed semi-quantitative RT-PCR using RNAs isolated from lymphoblasts issued from a healthy individual or from the proband. We used primers located in exon 11 (Ex11FLR), exon 12 (Ex12R), or on the overlapping junctions of exons 9–10 (Ex9-10For2) and exons 11q-12 (Ex11q-12R) [36] (Figure 5b). Compared to the estimated level of the full-length transcript (FL) in WT cells (considered equal to 100%) the patient had a (expected) reduction of 58.8% ± 4.8. The del11q isoform, almost undetectable in WT cells (5.1% ± 1.5 of total *BRCA1* transcripts), represents approximately half of the *BRCA1* RNAs expressed in cells from the patient (63.1% ± 2.7) (Figure 5c,d).

**Figure 6:**
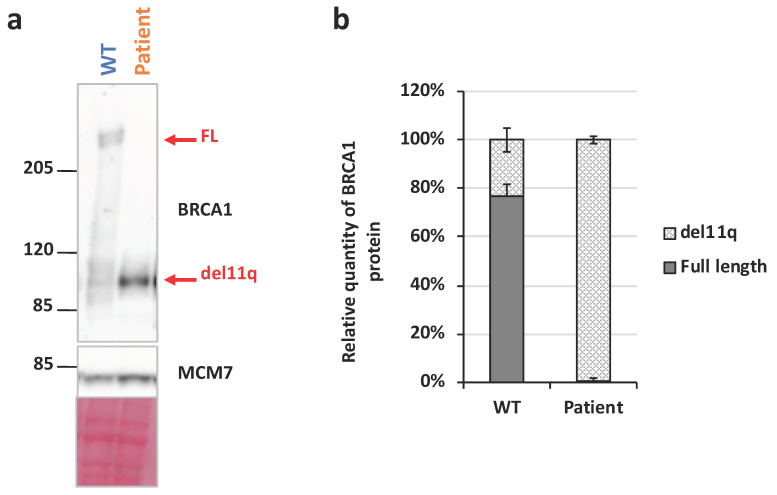
BRCA1 protein analysis. (**a**) Total protein extract from immortalized lymphoblasts from a control (WT) or the patient were loaded on 3–8% Tris-acetate SDS-PAGE gel and revealed with an anti-BRCA1 antibody recognizing the C terminal extremity. MCM7 is an internal loading control. (**b**) Relative amounts of BRCA1 protein isoforms from replicate experiments. (n = 7; mean ± SEM).

This result suggests that the presence of the c.791-_794del variant yields a strong use of the physiological alternative splice site at position c.787, resulting in an RNA encoding an in-frame deleted BRCA1 protein with normal C and N termini. Successive Western-blot analyses revealed the presence of the full length WT BRCA1 in the control’s cells and validated its absence in patient’s cells. The del11q protein is strongly expressed in the patient’s cells but almost undetectable in the control’s extracts (Figure 6a,b).

Therefore, the RT-PCR and Western-blot analysis support the fact that the c.797_794del *BRCA1* allele identified in the patient is transcribed and translated into a shorter protein and that the patient’s cells are totally devoid of the full length BRCA1 protein.

### 2.5. DNA Damage Response (DDR) Activation After Genotoxic Agents

Genotoxic agents like aphidicolin (Aph), hydroxyurea (HU) or MMC, inhibit, respectively, replicative polymerase and ribonucleotide reductase or create DNA interstrand crosslinks (a barrier to replication fork progression) leading to FANC/BRCA pathway activation. This pathway is composed of three groups of proteins: the FANCcore complex that mediates the monoubiquitination of the second group of components, FANCD2/FANCI, which form chromatin associated foci required for the recruitment and action of the proteins of the third group. This last group, which includes BRCA1, BRCA2, and the recombinase RAD51, participates in HR allowing for the protection and the resumption of arrested replication forks [37,38].

**Figure 7:**
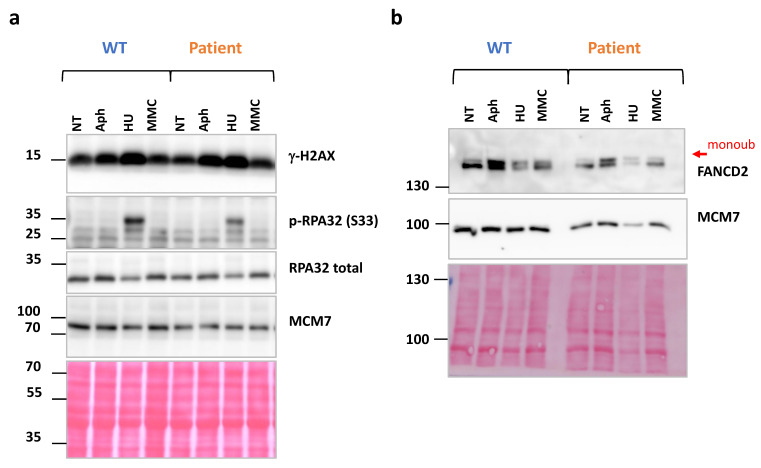
DDR and FANC/BRCA pathway activation after genotoxic agents’ overnight treatment. (**a**) Detection of DSB signaling by γ-H2AX, DNA resection revealed by p-RPA32 expression (p-RPA/RPA total) in patient and control cells after overnight treatment. (**b**) FANC/BRCA pathway activation revealed by FANCD2 monoubiquitination. (Aph: Aphidicolin at 0.6 µM; HU: Hydroxyurea at 5 mM or MMC: mitomycin C at 200 ng/mL).

We treated cells overnight with the replication inhibitors 0.6 µM aphidicolin (Aph) or 5 mM hydroxyurea (HU) or 200 ng/mL MMC and determined DNA damage and DNA damage response (DDR). We monitored phosphorylated histone H2AX (γ-H2AX), which marks double-strand breaks (DSB), phosphorylated replication protein A (p-RPA-S33), which marks single-strand DNA (ssDNA), as well as FANCD2 monoubiquitination, readout of the FANCcore complex activation. No significant difference was observed between patient and WT cells in the phosphorylation levels of H2AX and RPA and in the FANCD2 monoubiquitination (Figure 7a,b), demonstrating a normal activation of the early steps of DDR and of the FANC/BRCA pathway in the patient.

**Figure 8:**
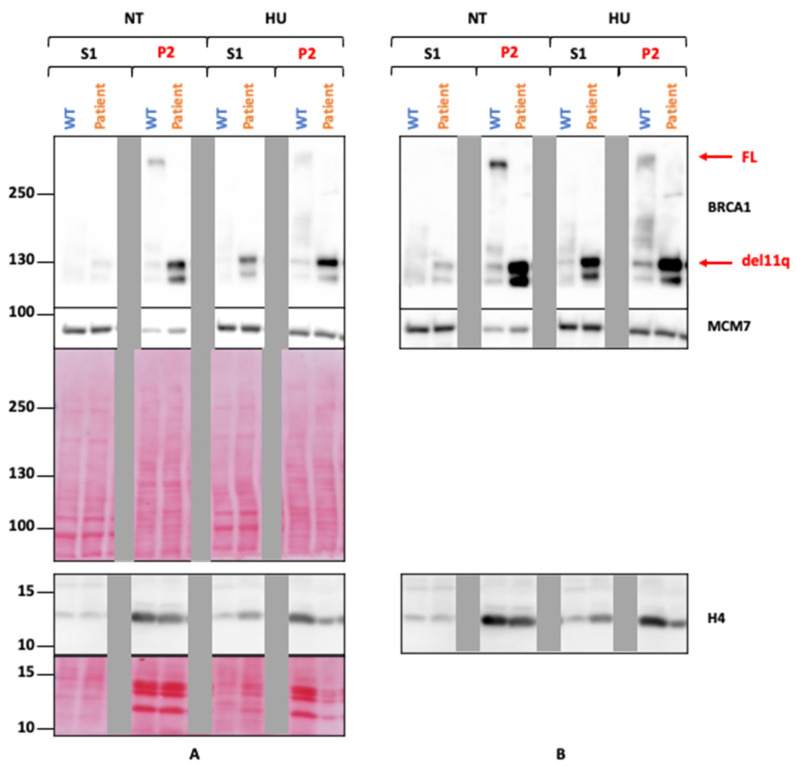
BRCA1 recruitment on chromatin. Soluble (S1) and chromatin fractions (P2) were analyzed by Western-blot (6% and 15% Tris-Glycine SDS-PAGE), after two culture conditions (treated with HU at 5 mM overnight versus untreated). An enrichment of histone H4 in chromatin extracts confirmed the fractionation. Gray bands mask a sample that cannot be publicly presented (**A**,**B**) in different acquisition times).

Wang et al. demonstrated that the del11q isoform of the BRCA1 protein maintains an average of 50% DNA damage repair capacity and interacts, although less efficiently, with the functional protein partners of full-length BRCA1 such as PALB2, BRCA2, RAD51 and CtIP [35]. In agreement with the maintenance of some repair activity, the del11q BRCA1 isoform (the only isoform detected in patient’s cells) is enriched in chromatin after genotoxic treatment and DDR activation, as observed in chromatin extracts (Figure 8).

**Figure 9:**
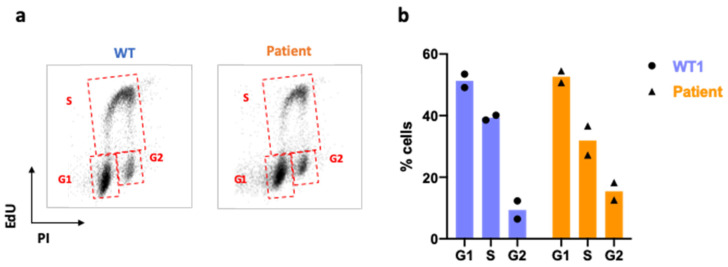
Cell cycle: (**a**) cell cycle determined by flux cytometry for each individual by EdU and propidium iodide (PI) staining. (**b**) Cell distribution by cycle phases (n = 2; mean).

Finally, in the absence of an exogenous stress, the cell cycle profile, i.e., the G1-S-G2/M distribution of the cell in the different phases, is similar between the control’s and the patient’s cells (Figure 9).

## 3. Discussion

We describe for the first time a patient presenting an isolated DOR, followed by resumptive POI, linked to compound heterozygous frameshift *BRCA1* mutations. The cellular hypersensitivity to the PARP inhibitor Olaparib and the chromosomal hypersensitivity to MMC clearly support a significant loss of the DNA repair capability of BRCA1 in the patient and are compatible with a diagnosis of FA [11,12,13]. However, at the time of the analysis, the patient did not show any typical clinical signs of FA (dysmorphia, microcephaly, BMF, anemia, thrombocytopenia, or congenital abnormalities), apart from the presence of café-au-lait spots that are not specific, and no tumor apart from an adrenocortical adenoma which is not a canonical Fanconi-associated tumor. Obviously, the patient is still a teenager and might develop BMF and cancers later in life.

The sensitivity of the patient’s cells to Olaparib and MMC exposure indicates that the identified *BRCA1* mutations affect the proficiency of the DNA process supporting a role of BRCA1 in oocyte DDR. Meiotic recombination occurs through the programmed formation of double-strand breaks (DSBs) in chromosomal DNA, a subset of such DSBs will progress into crossovers generating chromatids that mix the mother’s genetic information with that of the father, increasing genomic variation of the potential offspring. Oocytes repair meiotic DSBs by recruiting on the chromosomes the downstream repair factors BRCA1 and 53BP1 during meiosis I [39]. Moreover, beyond its activity on DNA, DSB repair by HR, and in DNA replication fork protection [37,38], it has been reported that BRCA1 exerts pivotal functions in spindle assembly, alignment of chromosomes, and spindle checkpoint regulation during mouse oocyte meiotic maturation [40]. Our results suggest that ovarian development depends on the normal repair of double-stranded DNA breaks that occur at recombination during meiosis. These results highlight an emerging concept of a critical role for major DNA repair genes (e.g., *BRCA2* and, as reported here, *BRCA1*) in ovarian development and function highlighted by mutations causing POI and here DOR [9,17,19].

Few patients with FA survive up to adulthood and in these cases genital malformations and hypoplastic gonads have been described in males and POI in female patients as found in female mouse models [15]. The DNA repair function of BRCA1 is considered essential for embryonic development and animal models have shown that a *BRCA1* loss-of-function results in early embryonic lethality [41,42]. Nevertheless, rare individuals with BRCA1 biallelic (hypomorphic) mutations can survive and present severe FA phenotype [43,44,45,46,47]. More unexpected, individual with biallelic mutations of *BRCA1* without cancer nor clinical FA-like features were also reported. Two healthy and fertile individuals 40 and 58-year-old, i.e., without any cancer and with two children at the time of the inclusion, carrying a homozygous donor splice site mutation in intron 11 (c.4096 + 3A > G) were identified during an analysis of breast and ovarian cancer families [48,49]. A homozygous c.3082C > T (p.Arg1028Cys) *BRCA1* mutation was reported in a patient, mother of two children, that developed a chronic lymphatic leukemia at 48 years without sign of FA or DNA damage hypersensitivity [50]. *BRCA1* compound heterozygous mutations in exon 11 (c.4065-4068del–p.Asn1355Lysfs*10) and in intron 22 (c.5406 + 7A > G–p.Asp1778Glyfs*27) were reported in another patient, mother of a girl, that developed ovarian and breast cancers at 43 and 44 years, without any FA-like symptoms [51]. However, in these studies, no MMC sensibility test was performed in the patients’ cells [48,49,50,51].

We have shown that, in our patient, a shorter BRCA1 protein is detected, certainly corresponding to the mutated c.791_794del allele, by analogy with Wang’s results [35]. The absence of clinical signs of FA suggests a protective mechanism that counteracts the functional defect of BRCA1. Thus, it is tempting to speculate that the BRCA1-del11q protein present in the patient’s, although unable to respond properly to DNA damage, probably maintains other functions of BRCA1 in the control of the activity/expression of several proteins via its E3-ubiquitin ligase activity or in the regulation of RNA transcription or translation [52,53,54,55] that might help to attenuate the pathological consequences of the c.791_794del mutation. Alternatively, the patient’s genetic background could be involved in reducing the consequences of BRCA1 loss of function, as shown recently: individuals with cancer pathogenic variants may be at a less elevated risk of cancer in the absence of a first-degree family history [56] or in the presence of protective common genetic variants that contribute to incomplete penetrance [57,58].

In the presence of heterozygous *BRCA1* pathogenic variants (PV), but without associated clinical signs of FA, and in the absence of a familial history of breast or ovarian cancers, an assessment of the actual oncological risk in this patient is difficult. These have already been reported in FA patients in the usual context of *BRCA2* or *FANC*x pathogenic variants, and with associated clinical abnormalities [59]. In the absence of reliable data on oncological risks, the patient’s follow-up strategy was discussed within a multidisciplinary team, including oncologists and geneticists. It was proposed to follow monitoring procedures similar to those for usual FA patients [60]. Hematological monitoring with basal bone marrow aspirate and complete blood count (BCC) (then BCC every 3 or 4 months) has been proposed to allow for the proactive monitoring of progressive cytopenias and myelodysplastic syndrome. Since the genetic diagnosis, the patient benefited from regular evaluations with an otolaryngologist alternating with oral self-examinations, a gynecologist, a proctologist, and a dermatologist, at least once a year and annual breast magnetic resonance imaging (MRI) was recommended from the age of 20.

In conclusion, our observation is the first implication of bi-allelic *BRCA1* mutations in isolated ovarian dysfunction or infertility/hypofertility in humans, without clinical signs of FA, highlighting the importance of BRCA1 in ovarian development and function.

This observation strengthens the new links between genes responsible for POI/DOR and cancer predisposition genes, and makes the genetic diagnosis of any unexplained DOR or POI essential. The identification of a POI/DOR etiology may have broader consequences than initially anticipated. Any unexplained DOR/POI must benefit from a genetic diagnosis including DNA repair genes, the main family responsible for POI [7]. Chromosomal fragility might be a useful screening tool in the evaluation of patients with unexplained POI/DOR and may reveal defects in double strand DNA repair. The expression of a truncated protein and/or the genetic background of the patients can reduce or delay the consequence of the identified mutations. The identification of this subpopulation of patients at risk is a medical priority. It might profoundly modify the management of these patients and their families. Despite the absence of somatic signs, chromosomal fragility equivalent to that of FA must lead to long-term follow-up within a multidisciplinary team, in particular with an oncogeneticist, in order to inform the patient of possible oncological risks, to offer monitoring and organize family genetic counseling.

## 4. Materials and Methods

### 4.1. Genetics Studies

A custom-made targeted NGS including all known genes (88) involved in POI to date was performed in the patient as previously described [7]. Another panel was used in the Gustave Roussy Institute comprising the following genes: *ACD* (*NM_001082486.1*; *exon 1*), *APC* (*NM_000038.5*), *ATM* (*NM_000051.3*), *BAP1* (*NM_004656.3*), *BARD1* (*NM_000465.3*), *BRCA1* (*NM_007294.3*), *BRCA2* (*NM_000059.3*), *BRIP1* (*NM_032043.2*), *BRK1* (*NM_018462.4*; *exon 2 and 3*), *CDH1* (*NM_004360.4*), *CDK4* (*NM_000075.3*; *exon 2*), *CDKN2A/p14* (*NM_058195.3*), *CDKN2A/p16* (*NM_000077.4*), *CHEK2* (*NM_007194.3*), *EPCAM* (*NM_002354.2*; *exon 8 and 9*), *FH* (*NM_000143.3*), *FLCN* (*NM_144997.6*), *MC1R* (*NM_002386.3*), *MET* (*NM_001127500.2*), *MITF* (*NM_000248.3*; *exon 9*), *MLH1* (*NM_000249.3*), *MRE11A* (*NM_005591.3*), *MSH2* (*NM_000251.2*), *MSH6* (*NM_000179.2*), *MUTYH* (*NM_001048171.1*), *NBN* (*NM_002485.4*), *PALB2* (*NM_024675.3*), *PMS2* (*NM_000535.6*; *exons 6 to 10*), *POLD1* (*NM_002691.3*; *exons 8 to 12*), *POLE* (*NM_006231.3*; *exons 9 to 14*), *POT1* (*NM_015450.2*), *PTEN* (*NM_000314.6*), *RAD51B* (*NM_133509.3*), *RAD51C* (*NM_058216.2*), *RAD51D* (*NM_002878.3*), *SDHB* (*NM_003000.2*), *STK11* (*NM_000455.4*), *TERF2IP* (*NM_018975.3*), *TERT* (*NM_198253.2*; *promoter*), *TP53n* (*NM_000546.5*), *VHL* (*NM_000551.3*), *XRCC2* (*NM_005431.1*) [23].

Briefly, NGS was performed using a library designed to capture all exons ±50 bp (Capture Agilent SureSelect QXT), then run on a MiSeq Illumina to a minimal depth of 100×. Sequencing data (FastQ files) were generated by MiSeq Analysis software v4.1.0 and subsequently, alignment (GRCh37) and variant calling (including structural variants) were performed with an in-house developed bioinformatics pipeline including BWA alignment, haplotype-based GATK variant calling, and snpEff annotation. Variants interpretation was performed following the standards and guidelines recommended by the American College of Medical Genetics (ACMG) [6] and by board-certified (Agence de la biomédecine, France) clinical molecular geneticists using the VarSome website (https://varsome.com, accessed on 9 June 2023).

To exclude the presence of other relevant variant(s) that could explain the ovarian phenotype of the patient, we performed WES on genomic DNA extracted from the peripheral blood of the patient. Library preparation, exome capture, sequencing, and data processing were performed by IntegraGen SA (Evry, France), according to their in-house procedures. A data analysis was performed as described previously [7,61,62]. In brief, exon enrichment was performed on 200 ng of DNA, using the Agilent SureSelect Human All Exons kit version CRE (Agilent Technologies, Santa Clara, CA, USA). Exon-enriched libraries were subjected to 75 bp paired-end sequencing on a HiSeq2500, according to the manufacturer’s protocol. Read alignment to the human reference genome (GRCh37) and variant calling were performed using the Illumina pipeline (CASAVA 1.8.2). The metrics and quality score of the exome output are specified in Table 3. A variant annotation was performed using the Ensembl’s Variant Effect Predictor. The variants were filtered using SIRIUS, an IntegraGen in-house pipeline platform. The variants were processed using a combination of in silico bioinformatic predictors and bibliographic research (regarding gene function, expression, the animal model described, etc.).

The two pathogenic *BRCA1* variants detected in the patient were confirmed via direct genomic Sanger sequencing using the followings primers pairs: for exon 8, 5′-ctcccagccccagacatttt-3′ and 5′-aggtgggaactgcgtctttt-3′, and for exon 11, 5′-ggtgtggtttctgcataggga-3′ and 5′-agcccatctgttatgttggct-3′.

### 4.2. Chromosomal Analysis

Chromosomal breakage studies were performed in cultures of peripheral lymphocytes obtained from the patient and two healthy women as controls. The experiments were performed at the Gustave Roussy following a standard in-house protocol [7,9,16]. Fresh peripheral blood lymphocytes were cultured under standard conditions for karyotyping. DNA damage was induced by treating the cells with mitomycin C (MMC; Sigma-Aldrich, Saint Quentin Fallavier, France) for 48 h to examine cellular hypersensitivity to DNA crosslinking agents. Three treatment conditions were used without MMC to analyze spontaneous DNA damage and 150 nM MMC. Chromosome breaks were scored by an experienced cytogeneticist using at least 20 metaphases.

### 4.3. Cell Culture

The lymphoblasts from the patient were immortalized by EBV in Généthon (Evry, France) and were cultured in RPMI + 20% FBS + 100 μg/mL streptomycin + 100 U/mL penicillin (Gibco, Thermo Fisher Scientific, Illkirch-Graffenstaden, France).

### 4.4. Drugs Treatments on Cell Cultures

The genotoxic agents used were: Mitomycin C (MMC, Sigma M0503), Olaparib (AZD2281, S1060, Selleckchem, Euromedex, Souffelweyersheim, France), Aphidicolin (Aph, Sigma A0781) and Hydroxyurea (HU, Sigma, H8627).

### 4.5. Survival Test

Cells are plated at 100.000 cells per P24 well, with different doses of MMC or Olaparib. Three days later, the live cell number is detected. The cell survival percentage is relatively determined with no treated cells.

### 4.6. Transcripts Analysis

*BRCA1* transcripts were evaluated by RT-PCR. The total amount of RNA was extracted using the Maxwell RSC simplyRNA Cells Kit (Promega, Charbonnières-les-Bains, France, AS1390) and reverse-transcribed using the RevertAid First Strand cDNA Synthesis Kit (Thermo Scientific, Illkirch-Graffenstaden, France, K1622). PCR was performed on cDNA using the DreamTaq DNA polymerase (Thermo Scientific, #EP0713) and 30 cycles (95 °C for 30 s, 50 °C for 30 s, 72 °C for 1 min). The following primers (Eurogentec) were used for PCR: Ex9-10For2 (junction exons 9 and 10: forward, 5′-CAACTTATTGCAGTGTGGGAGA-3′); Ex11FLR (in exon 11 after the alternative donor site: reverse 5′-GGAGTCCGCCTATCATTACATG-3′); Ex12R (in exon 12: reverse 5′-AATGTCACTCTGAGAGGATAGC-3′); Ex11q-12R (junction exons del11q and 12: 5′-CCAGATGCTGCTTCACCCT-3′) (Figure 4b) [36]. Amplicons were analyzed on 2% agarose gel in TBE.

### 4.7. Protein Analysis by Western-Blot

Whole-cell extracts were prepared via resuspension of cellular pellet cells in the lysis buffer (50 mM of Tris-HCl pH 7.5, 20 mM of NaCl, 1 mM of MgCl_2_, 0.1% SDS, 0.1% benzonase (Novagen, Merk), cOmplete EDTA-free protease inhibitor cocktail (Roche, Sigma-Aldrich) and anti-phosphatase cocktail (PhosSTOP, Roche, Sigma-Aldrich) for 20 min at room temperature. Protein concentrations were determined via the Bradford method (Bio-Rad Protein assay, Bio-Rad, Marnes-la-Coquette, France, 5000006). Samples were combined 4× with the Laemmli buffer containing 10% β-mercaptoethanol and were denatured at 55 °C for BRCA1 (Figure 5) or 98 °C for other proteins. Proteins (50 μg) were separated by SDS-PAGE in 3–8% Tris-acetate gel (EA03755, Invitrogen, Toulouse, France) to detect BRCA1 or in 12% Tris-glycine SDS-PAGE for other proteins. Proteins were semi-dried and transferred to nitrocellulose membranes for 90 min at 20 V or in the iBlot^®^2 gel transfer (ThermoFisher Scientific, Waltham, MA, USA) for 15 min at 20 V in 3–8% Tris-acetate gel. Blots were probed with the following antibodies against: BRCA1 (SC-6954, Santa-Cruz, Heidelberg, Germany), MCM7 (Ab2360, Abcam, Amsterdam, The Netherlands), FANCD2 (FI-17, Santa-Cruz), p-RPA32(S33) (A300-246A, Bethyl, Ozyme, Saint-Cyr-l’Ecole, France), RPA32 (Ab16855, Abcam), γ-H2AX (05-636, Millipore, Merk, Guyancourt, France), and Histone H4 (Ab31830, Abcam). The proteins were visualized using an enhanced chemiluminescence system (Western Bright ECL, Advansta, Diagomics, Blagnac, France). All Western blot quantifications were performed with the ImageJ software (version: 2.0.0-rc-68/1.52p) using densitometry measures obtained from ImageQuant LAS 4000 (GE Healthcare Life Science, Courtabeauf, France).

### 4.8. Chromatin Extracts

Cell pellets were resuspended in solution A (Hepes pH 7.9 10 mM, MgCl_2_ 1.5 mM, sucrose 12%, glycerol 10%, DTT 1 mM, protease, and phosphatase inhibitors) supplemented with 0.1% Triton X100. After 5 min of incubation on ice, the cells were centrifuged for 4 min at 1300× *g*, and the soluble proteins (S1) were collected. The nuclei were incubated for 10 min on ice in solution B (EDTA 3 mM, EGTA 0.2 mM, DTT 1 mM, protease, and phosphatase inhibitors) and then centrifuged for 4 min at 1700× *g*. The chromatin (P2) was resuspended in solution B then centrifuged for 1 min at 10,000× *g*. The S1 and P2 fractions were mixed in lysis buffer. All fractions were denatured by boiling and analyzed by Western blotting on 6% and 15% SDS-PAGE gels as described above.

### 4.9. Cell Cycle Studies

Cells were incubated with 10 µM EdU for 30 min then cellular pellet was resuspended in cold PBS and fixed with cold 70% ethanol for a minimum of 3 h. After two washes by PBS, cellular pellet was incubated for 60 min with azide solution according to Click-iT^®^ EdU imaging kit protocol (C10338, Molecular Probes Life, Thermo Fisher Scientific, Illkirch-Graffenstaden, France). After one wash with PBS-5% BSA-0.25% triton, DNA was stained for 20 min with a solution containing 1 mg/mL propidium iodide (PI) and 32 mg/mL RNAse A in PBS. After a wash with PBS-5% BSA-0.25% triton, cellular staining was detected by flux cytometry with a BD Accuri™ C6 flow cytometer.

## Figures and Tables

**Table 1:** Clinical and biological data of the patient.

Age	14 yr 3 m	15 yr	15 yr 1 m	16 yr 8 m to 17 yr	17 yr 4 m	18 yr	18 yr 3 m	18 yr 6 m	18 yr 6 m to 18 yrs 10 m
**Menses**	Secondary amenorrhea due to an adrenal corticotrop adenoma	Spontaneous regular menses (Day 3 of the cycle)		Amenorrhea, hot flushes	Day 3 bleeding induced by progestin	Spontaneous regular menses (Day 3 of the cycle cycle)			Frequent menstrual bleeding every 2 weeks before spontaneous pregnancy
**FSH UI/L**	0.1	11.2	9.3	49	25	9.4	7.3	10.5	NP
**LH UI/L**	0.1	12.8	2.9	14	8.4	3.9	5.2	8.9	NP
**Estradiol pg/mL**	38	96	30	NP	52	57.7	31	45.5	NP
**AMH** **ng/mL**	0.10	0.10	NP	0.1	0.22	0.15	0.3	NP	NP
**Testosterone nmol/L**	23	0.99	NP	NP	0.91	NP	NP	0.59	NP

yr = years; m = months; NP: not performed; FSH expressed in UI/L (normal ranks follicular phase: 3.03–8.08); LH expressed in UI/L (normal ranks follicular phase: 1.8–11.8); estradiol expressed in pg/mL (normal ranks follicular phase: 21–250); AMH expressed in ng/mL (normal ranks 0.6–7.4), testosterone expressed in nmol/L (normal ranks for tanner 5 women: 0.27–1.5).

**Table 2:** Chromosomic fragility in response to mitomycin C revealed by the percentage of aberrant metaphases and radial figures, and number of breaks per metaphases obtained in patient’s or control’s cells (WT1, WT2).

	Aberrant Metaphases		Radial Figures		Breaks/Metaphase	
	Untreated	MMC (150 nM)	Untreated	MMC (150 nM)	Untreated	MMC (150 nM)
**Patient**	4/50 (8%)	48/50 (96%)	0/50 (0%)	Numerous	0.08	8.74
**WT 1**	0/50 (0%)	1/50 (2%)	0/50 (0%)	0/50 (0%)	0	0.02
**WT 2**	0/50 (0%)	7/50 (14%)	0/50 (0%)	2/50 (4%)	0	0.24

**Table 3:** Metrics of the whole exome sequencing performed for the patient.

WES Metrics	The Patient
**Gbases**	5.19
**Number of reads (millions)**	25.94
**% Alignment**	97.97
**% Mismatch Rate R1**	0.28
**% Mismatch Rate R2**	0.58
**% ≥Q30 bases**	96.2
**Mean Quality Score**	36
**Mean Depth (X)**	59.17
**% of bases covered at 25×**	97.7

## Data Availability

Original data generated and analyzed during this study are included in this published article or in the data repositories listed in the References.

## References

[1] World Health Organization (2023). Infertility Prevalence Estimates, 1990–2021.

[2] Webber L., Davies M., Anderson R., Bartlett J., Braat D., Cartwright B., Cifkova V., de Muinck Keizer-Schrama S., Hogervorst E., Janse F. (2016). ESHRE Guideline: Management of Women with Premature Ovarian Insufficiency. Hum. Reprod..

[3] Huhtaniemi I., Hovatta O., La Marca A., Livera G., Monniaux D., Persani L., Heddar A., Jarzabek K., Laisk-Podar T., Salumets A. (2018). Advances in the Molecular Pathophysiology, Genetics, and Treatment of Primary Ovarian Insufficiency. Trends Endocrinol. Metab..

[4] Bidet M., Bachelot A., Bissauge E., Golmard J.L., Gricourt S., Dulon J., Coussieu C., Badachi Y., Touraine P. (2011). Resumption of Ovarian Function and Pregnancies in 358 Patients with Premature Ovarian Failure. J. Clin. Endocrinol. Metab..

[5] Alviggi C., Andersen C.Y., Buehler K., Conforti A., De Placido G., Esteves S.C., Fischer R., Galliano D., Polyzos N.P., Sunkara S.K. (2016). A New More Detailed Stratification of Low Responders to Ovarian Stimulation: From a Poor Ovarian Response to a Low Prognosis Concept. Fertil. Steril..

[6] Richards S., Aziz N., Bale S., Bick D., Das S., Gastier-Foster J., Grody W.W., Hegde M., Lyon E., Spector E. (2015). Standards and Guidelines for the Interpretation of Sequence Variants: A Joint Consensus Recommendation of the American College of Medical Genetics and Genomics and the Association for Molecular Pathology. Genet. Med..

[7] Heddar A., Ogur C., Da Costa S., Braham I., Billaud-Rist L., Findikli N., Beneteau C., Reynaud R., Mahmoud K., Legrand S. (2022). Genetic Landscape of a Large Cohort of Primary Ovarian Insufficiency: New Genes and Pathways and Implications for Personalized Medicine. eBioMedicine.

[8] Veitia R.A. (2020). Primary Ovarian Insufficiency, Meiosis and DNA Repair. Biomed. J..

[9] Caburet S., Heddar A., Dardillac E., Creux H., Lambert M., Messiaen S., Tourpin S., Livera G., Lopez B.S., Misrahi M. (2021). Homozygous Hypomorphic *BRCA2* Variant in Primary Ovarian Insufficiency without Cancer or Fanconi Anaemia Trait. J. Med. Genet..

[10] Alter B.P., Rosenberg P.S., Brody L.C. (2006). Clinical and Molecular Features Associated with Biallelic Mutations in FANCD1/BRCA2. J. Med. Genet..

[11] Bogliolo M., Surrallés J. (2015). Fanconi Anemia: A Model Disease for Studies on Human Genetics and Advanced Therapeutics. Curr. Opin. Genet. Dev..

[12] Niraj J., Färkkilä A., D’Andrea A.D. (2019). The Fanconi Anemia Pathway in Cancer. Annu. Rev. Cancer Biol..

[13] Fiesco-Roa M.O., Giri N., McReynolds L.J., Best A.F., Alter B.P. (2019). Genotype-Phenotype Associations in Fanconi Anemia: A Literature Review. Blood Rev..

[14] Gueiderikh A., Maczkowiak-Chartois F., Rouvet G., Souquère-Besse S., Apcher S., Diaz J.-J., Rosselli F. (2021). Fanconi Anemia A Protein Participates in Nucleolar Homeostasis Maintenance and Ribosome Biogenesis. Sci. Adv..

[15] Tsui V., Crismani W. (2019). The Fanconi Anemia Pathway and Fertility. Trends Genet..

[16] Fouquet B., Pawlikowska P., Caburet S., Guigon C., Mäkinen M., Tanner L., Hietala M., Urbanska K., Bellutti L., Legois B. (2017). A homozygousFANCMmutation Underlies a Familial Case of Non-Syndromic Primary Ovarian Insufficiency. eLife.

[17] Weinberg-Shukron A., Rachmiel M., Renbaum P., Gulsuner S., Walsh T., Lobel O., Dreifuss A., Ben-Moshe A., Zeligson S., Segel R. (2018). Essential Role of *BRCA2* in Ovarian Development and Function. N. Engl. J. Med..

[18] Qin Y., Zhang F., Chen Z.-J. (2019). BRCA2 in Ovarian Development and Function. N. Engl. J. Med..

[19] Turchetti D., Zuntini R., Tricarico R., Bellacosa A. (2019). BRCA2 in Ovarian Development and Function. N. Engl. J. Med..

[20] Hu K.-L., Wang S., Ye X., Zhang D. (2020). Effects of BRCA Gene Mutation on Female Reproductive Potential: A Systematic Review. Maturitas.

[21] Vanni V.S., Campo G., Cioffi R., Papaleo E., Salonia A., Vigano P., Lambertini M., Candiani M., Meirow D., Orvieto R. (2022). The Neglected Members of the Family: Non-BRCA Mutations in the Fanconi Anemia/BRCA Pathway and Reproduction. Hum. Reprod. Update.

[22] Scully R., Chen J., Plug A., Xiao Y., Weaver D., Feunteun J., Ashley T., Livingston D.M. (1997). Association of BRCA1 with Rad51 in Mitotic and Meiotic Cells. Cell.

[23] Baz M., Gondran-Teiller V., Bressac B., Cabaret O., Fievet A., Dimaria M., Goldbarg V., Colas C., Bonnet-Dupeyron M.N., Tinat J. (2023). The Frequency of Germline BRCA and Non-BRCA HR-Gene-Variants in a Cohort of Pancreatic Cancer Patients. Dig. Dis. Sci..

[24] Tang N.L.S. (1999). Prevalence of Mutations in the BRCA1 Gene Among Chinese Patients With Breast Cancer. J. Natl. Cancer Inst..

[25] de la Hoya M., Pérez-Segura P., Van Orsouw N., Diaz-Rubio E., Caldés T. (2001). Spanish Family Study on Hereditary Breast and/or Ovarian Cancer: Analysis of theBRCA1 Gene. Int. J. Cancer.

[26] Vega A., Torres M., Martínez J.I., Ruiz-Ponte C., Barros F., Carracedo A. (2002). Analysis of BRCA1 and BRCA2 in Breast and Breast/Ovarian Cancer Families Shows Population Substructure in the Iberian Peninsula. Ann. Hum. Genet..

[27] Fernandes G.C., Michelli R.A.D., Galvão H.C.R., Paula A.E., Pereira R., Andrade C.E., Felicio P.S., Souza C.P., Mendes D.R.P., Volc S. (2016). Prevalence of *BRCA1/BRCA2* Mutations in a Brazilian Population Sample at-Risk for Hereditary Breast Cancer and Characterization of Its Genetic Ancestry. Oncotarget.

[28] Rebbeck T.R., Friebel T.M., Friedman E., Hamann U., Huo D., Kwong A., Olah E., Olopade O.I., Solano A.R., Teo S.-H. (2018). Mutational Spectrum in a Worldwide Study of 29,700 Families with *BRCA1* or *BRCA2* Mutations. Hum. Mutat..

[29] Oostra A.B., Nieuwint A.W.M., Joenje H., de Winter J.P. (2012). Diagnosis of Fanconi Anemia: Chromosomal Breakage Analysis. Anemia.

[30] Lord C.J., Ashworth A. (2017). PARP Inhibitors: Synthetic Lethality in the Clinic. Science.

[31] Michl J., Zimmer J., Tarsounas M. (2016). Interplay between Fanconi Anemia and Homologous Recombination Pathways in Genome Integrity. EMBO J..

[32] Portier L., Desterke C., Chaker D., Oudrhiri N., Asgarova A., Dkhissi F., Turhan A.G., Bennaceur-Griscelli A., Griscelli F. (2021). iPSC-Derived Hereditary Breast Cancer Model Reveals the BRCA1-Deleted Tumor Niche as a New Culprit in Disease Progression. Int. J. Mol. Sci..

[33] Li D., Harlan-Williams L.M., Kumaraswamy E., Jensen R.A. (2019). BRCA1—No Matter How You Splice It. Cancer Res..

[34] Raponi M., Douglas A.G.L., Tammaro C., Wilson D.I., Baralle D. (2012). Evolutionary Constraint Helps Unmask a Splicing Regulatory Region in BRCA1 Exon 11. PLoS ONE.

[35] Wang Y., Bernhardy A.J., Cruz C., Krais J.J., Nacson J., Nicolas E., Peri S., Van Der Gulden H., Van Der Heijden I., O’Brien S.W. (2016). The BRCA1-Δ11q Alternative Splice Isoform Bypasses Germline Mutations and Promotes Therapeutic Resistance to PARP Inhibition and Cisplatin. Cancer Res..

[36] Seo A., Steinberg-Shemer O., Unal S., Casadei S., Walsh T., Gumruk F., Shalev S., Shimamura A., Akarsu N.A., Tamary H. (2018). Mechanism for Survival of Homozygous Nonsense Mutations in the Tumor Suppressor Gene *BRCA1*. Proc. Natl. Acad. Sci. USA.

[37] Renaudin X., Rosselli F. (2020). The FANC/BRCA Pathway Releases Replication Blockades by Eliminating DNA Interstrand Cross-Links. Genes.

[38] Helbling-Leclerc A., Garcin C., Rosselli F. (2021). Beyond DNA Repair and Chromosome Instability—Fanconi Anaemia as a Cellular Senescence-Associated Syndrome. Cell Death Differ..

[39] Leem J., Kim J.S., Oh J.S. (2023). Oocytes Can Repair DNA Damage during Meiosis via a Microtubule-Dependent Recruitment of CIP2A–MDC1–TOPBP1 Complex from Spindle Pole to Chromosomes. Nucleic Acids Res..

[40] Xiong B., Li S., Ai J.-S., Yin S., OuYang Y.-C., Sun S.-C., Chen D.-Y., Sun Q.-Y. (2008). BRCA1 Is Required for Meiotic Spindle Assembly and Spindle Assembly Checkpoint Activation in Mouse Oocytes1. Biol. Reprod..

[41] Gowen L.C., Johnson B.L., Latour A.M., Sulik K.K., Koller B.H. (1996). Brca1 Deficiency Results in Early Embryonic Lethality Characterized by Neuroepithelial Abnormalities. Nat. Genet..

[42] Hohenstein P., Kielman M.F., Breukel C., Bennett L.M., Wiseman R., Krimpenfort P., Cornelisse C., Van Ommen G.-J., Devilee P., Fodde R. (2001). A Targeted Mouse Brca1 Mutation Removing the Last BRCT Repeat Results in Apoptosis and Embryonic Lethality at the Headfold Stage. Oncogene.

[43] Domchek S.M., Tang J., Stopfer J., Lilli D.R., Hamel N., Tischkowitz M., Monteiro A.N.A., Messick T.E., Powers J., Yonker A. (2013). Biallelic Deleterious *BRCA1* Mutations in a Woman with Early-Onset Ovarian Cancer. Cancer Discov..

[44] Sawyer S.L., Tian L., Kähkönen M., Schwartzentruber J., Kircher M., Majewski J., Dyment D.A., Innes A.M., University of Washington Centre for Mendelian Genomics, FORGE Canada Consortium (2015). Biallelic Mutations in *BRCA1* Cause a New Fanconi Anemia Subtype. Cancer Discov..

[45] Freire B.L., Homma T.K., Funari M.F.A., Lerario A.M., Leal A.M., Velloso E.D.R.P., Malaquias A.C., Jorge A.A.L. (2018). Homozygous Loss of Function BRCA1 Variant Causing a Fanconi-Anemia-like Phenotype, a Clinical Report and Review of Previous Patients. Eur. J. Med. Genet..

[46] Chirita-Emandi A., Andreescu N., Popa C., Mihailescu A., Riza A.-L., Plesea R., Ioana M., Arghirescu S., Puiu M. (2021). Biallelic Variants in *BRCA1* Gene Cause a Recognisable Phenotype within Chromosomal Instability Syndromes Reframed as BRCA1 Deficiency. J. Med. Genet..

[47] Keupp K., Hampp S., Hübbel A., Maringa M., Kostezka S., Rhiem K., Waha A., Wappenschmidt B., Pujol R., Surrallés J. (2019). Biallelic Germline *BRCA1* Mutations in a Patient with Early Onset Breast Cancer, Mild Fanconi Anemia-like Phenotype, and No Chromosome Fragility. Mol. Genet. Genom. Med..

[48] Byrjalsen A., Steffensen A.Y., Hansen T.v.O., Wadt K., Gerdes A.-M. (2017). Classification of the Spliceogenic *BRCA1* c.4096+3A>G Variant as Likely Benign Based on Cosegregation Data and Identification of a Healthy Homozygous Carrier. Clin. Case Rep..

[49] Arason A., Agnarsson B.A., Johannesdottir G., Johannsson O.T., Hilmarsdottir B., Reynisdottir I., Barkardottir R. (2019). The BRCA1 c.4096+3A>G Variant Displays Classical Characteristics of Pathogenic BRCA1 Mutations in Hereditary Breast and Ovarian Cancers, But Still Allows Homozygous Viability. Genes.

[50] Davide B., Francesca M., Valeria P., Irene F., Bernardo B. (2018). BRCA1 Homozygous Unclassified Variant in a Patient with Non-Fanconi Anemia: A Case Report. Oncol. Lett..

[51] Kwong A., Ho C.Y.S., Shin V.Y., Au C.H., Chan T.L., Ma E.S.K. (2021). A Case Report of Germline Compound Heterozygous Mutations in the BRCA1 Gene of an Ovarian and Breast Cancer Patient. Int. J. Mol. Sci..

[52] Takaoka M., Miki Y. (2018). BRCA1 Gene: Function and Deficiency. Int. J. Clin. Oncol..

[53] Witus S.R., Stewart M.D., Klevit R.E. (2021). The BRCA1/BARD1 Ubiquitin Ligase and Its Substrates. Biochem. J..

[54] Witus S.R., Zhao W., Brzovic P.S., Klevit R.E. (2022). BRCA1/BARD1 Is a Nucleosome Reader and Writer. Trends Biochem. Sci..

[55] Berthel E., Vincent A., Eberst L., Torres A.G., Dacheux E., Rey C., Marcel V., Paraqindes H., Lachuer J., Catez F. (2020). Uncovering the Translational Regulatory Activity of the Tumor Suppressor BRCA1. Cells.

[56] Jackson L., Weedon M.N., Green H.D., Mallabar-Rimmer B., Harrison J.W., Wood A.R., Ruth K.S., Tyrrell J., Wright C.F. (2023). Influence of Family History on Penetrance of Hereditary Cancers in a Population Setting. EClinicalMedicine.

[57] Downs B., Sherman S., Cui J., Kim Y.C., Snyder C., Christensen M., Luo J., Lynch H., Wang S.M. (2019). Common Genetic Variants Contribute to Incomplete Penetrance: Evidence from Cancer-Free BRCA1 Mutation Carriers. Eur. J. Cancer.

[58] Coignard J., Lush M., Beesley J., O’Mara T.A., Dennis J., Tyrer J.P., Barnes D.R., McGuffog L., Leslie G., Bolla M.K. (2021). A Case-Only Study to Identify Genetic Modifiers of Breast Cancer Risk for BRCA1/BRCA2 Mutation Carriers. Nat. Commun..

[59] Malric A., Defachelles A., Leblanc T., Lescoeur B., Lacour B., Peuchmaur M., Maurage C., Pierron G., Guillemot D., d’Enghien C.D. (2015). Fanconi Anemia and Solid Malignancies in Childhood: A National Retrospective Study. Pediatr. Blood Cancer.

[60] Walsh M.F., Chang V.Y., Kohlmann W.K., Scott H.S., Cunniff C., Bourdeaut F., Molenaar J.J., Porter C.C., Sandlund J.T., Plon S.E. (2017). Recommendations for Childhood Cancer Screening and Surveillance in DNA Repair Disorders. Clin. Cancer Res..

[61] Heddar A., Dessen P., Flatters D., Misrahi M. (2019). Novel STAG3 Mutations in a Caucasian Family with Primary Ovarian Insufficiency. Mol. Genet. Genom..

[62] Heddar A., Beckers D., Fouquet B., Roland D., Misrahi M. (2020). A Novel Phenotype Combining Primary Ovarian Insufficiency Growth Retardation and Pilomatricomas With MCM8 Mutation. J. Clin. Endocrinol. Metab..

